# Scoliosis – The current concepts

**DOI:** 10.4103/0019-5413.58600

**Published:** 2010

**Authors:** Dilip Kumar Sengupta, John K Webb

**Affiliations:** Assistant Professor, Department of Orthopaedic Surgery, Dartmouth-Hitchcock Medical Center, Lebanon, USA; 1 Consultant Spine Surgeon, Queens' Medical Centre, Nottingham, UK

We sincerely appreciate this editorial opportunity to present our thoughts on the changing concepts of scoliosis and other spinal deformities. The beginning of surgical correction of spinal deformity is marked with Paul Harrington introducing Harrington rod instrumentation in 1955.^1^ Since then a lot of progress has been made in various fields around spinal deformity management. Importance of early diagnosis has given birth to school screening, intrauterine detection of congenital spinal deformities, and genetic markers. The instrumentation technique progressed through generations from two-point fixation with H-rod, to segmental fixation using hooks with CD instrumentation or sublaminar Luque wiring, spinal and vertebral derotation using pedicle screws, anterior instrumentation, and a combination of any of these techniques. This has further developed into pelvic fixation for neuromuscular deformities, spinal osteotomies for rigid and severe deformities in post-tubercular patients and in adult scoliosis, and growing rods in infants permitting spinal growth. Evolution of neuromonitoring permits more aggressive deformity correction surgeries. Spinal deformity surgery is rightly considered ‘Cinderella’ in spinal surgery. However, we are still locked in the fact that the deformity surgery converts a physiological mobile spine into a long, rigid, fused spinal segment like a femur! Ideal goal of treatment should be correction of the deformity, but leave it mobile and functional. We are not even close to achieving that goal!

From the management point of view, spinal deformities may be broadly classified according to the age-group, pediatric or adult deformity, or according to the predominant plane of deformity, coronal (scoliosis) or sagittal (kyphosis). Infection, particularly tuberculosis, may lead to a very severe and rigid spinal deformity, across the age group, and forms the bulk of the deformity surgery in the East. Natural history and management of this problem is very different from deformities of other etiologies, like congenital, idiopathic, syndromal or neuro-muscular scoliosis.

Congenital scoliosis has been comprehensively classified by McMaster.^2^ This may lead to a short sharp curve, which may rapidly progress depending on the type of congenital anomaly. MRI scan and ultrasound may provide intra-uterine detection of the deformity.^3^ If the deformity is progressive, an early short segment fusion may be the ideal form of treatment. Infantile idiopathic scoliosis (IIS) is more common in northern Europe.^4^ Most infants resolve their scoliosis with growth, while in others the deformity may undergo a very rapid, malignant curve progression. The key to successful treatment of IIS is early recognition of a progressive curve, which may be selected for further treatment early enough. Mehta described rib-vertebral angle difference (RVAD) and overlapping of the rib head on the vertebral body as crucial radiological indicator of a progressive curve.^5^ The second problem in surgical treatment IIS is allowing spinal growth in the instrumented segment. Convex hemiepiphyseodesis alone does not hold the curve progression, and requires posterior instrumentation as well.^6^ Posterior surgery must be done with experiosteal dissection to prevent inadvertent fusion. Various instrumentation techniques have been described to accommodate spinal growth. Conventional growing rods, involves dual rods and a connector, and periodic intervention for rod elongation. This is associated with frequent complications.^7^ Webb described Luque-Trolley which does not require periodic intervention; the system may be replaced with definitive posterior instrumentation in adolescence. Some of these patients may not even need any definitive surgery in adolescence!^8^ In the current focus issue of Indian Journal of Orthopaedics, Teli *et al*.^9^ described an interesting alternative to the growing rod technique. In the surgical treatment of all Early-onset Idiopathic Scoliosis (EIS) in children under 10 years, anterior growth arrest by epiphyseodesis should be considered together with posterior instrumented fusion to prevent crank-shaft phenomenon. Canavese *et al*. presented the effect of dorsal arthrodesis on spinal growth, and thoracic dimensions in immature rabbit model.^10^

Unnikrishnan *et al*.^11^ discussed their 25 years of experience treating Adolescent-onset Indiopathic Scoliosis (AIS) in the current issue. Initial management in AIS (10 years or older) is essentially conservative, with periodic observation, exercise or bracing, until the curve reaches a certain magnitude. The threshold for surgery varies around 35 to 45°, depending on the age, and rapidity of progression. The aim is to stop the curve progressing unabated into adult life, which may create cosmetic problem in the short term, and cardio-respiratory complications and/or back pain in the adult life. Wong and Tan^12^ has discussed the natural history of scoliosis, school screening program for early diagnosis, and genetic markers in his review article in the current focus issue. The need for MRI scan as a routine pre-operative investigation for AIS is not beyond debate. Rajasekaran *et al*.^13^ has discussed this topic in the forthcoming focus issue.

Do physical therapy and bracing play any role in the treatment of scoliosis? There is level II evidence supporting the view that brace effectively slows down curve progression during the rapid growth phase in adolescence.^14^ But, there is no data in support of the view that brace or exercise can alter the natural history of scoliosis permanently.^15^

If a curve-progression can be predicted early, there may be a great advantage with early surgery, which offers better correction, with shorter fusion. Once the curve becomes severe and rigid, it requires longer fusion, with or without an anterior release. Fusion of the thoracic spine may not affect spinal motion significantly, but when the fusion needs to be extended into the lumbar spine, particularly L3 or lower, it affects spinal biomechanics significantly. The deleterious effect of a long fusion stopping over two or three remaining lumbar motion segments may lead to painful disc degeneration in early adult life, which poses a very difficult problem to solve.

The principle aim of surgical correction of scoliosis is to achieve balance. A relatively large curve, when appropriately balanced with an equally large compensatory curve, bringing the head over pelvis, may frequently go into adult life with minimal symptom. The goal, therefore, should be fusion of a minimal segment to achieve a balanced and straight spine. This has given birth to the concept of selective fusion of a primary/major curve, leaving the compensatory/minor curve alone. A too-short fusion may lead to ‘add on’ of a vertebra beyond the end vertebra, where as too-long fusion or too much correction of the selective curve may cause decompensation of a previously compensated scoliosis. Whether a few degrees of better curve correction matter in the long run is debatable.^16^ Wajanavisit *et al*.^17^ discuss their methods of selective short segment fusion for thoracic curves in this focus issue.

Planning the strategy of selective fusion without losing balance requires a thorough understanding of the deformity, as well as clear understanding of the definitions of primary vs. compensatory curves, major vs. minor curves, and structural vs. non-structural curves. King *et al*.^18^ reviewed the results of selective thoracic fusion and proposed the classification of scoliosis predominantly affecting the thoracic spine. King classification is easier to understand and covers most of the common scoliosis cases, but fails to include the lumbar curves. Lenke^19^ described a more comprehensive classification of scoliosis, which includes lumbar curves as well, and separates structural from non-structural types in the secondary or compensatory curves, and takes into account the thoracic hypo-kyphosis and lumbar modifiers.

The basic principles of scoliosis surgery were proposed nearly four decades ago by Goldstein^20^ and Moe.^21^ These include; fuse all the vertebrae within the primary/major curve, fuse from cephalad neutral to caudal neutral vertebra, and caudal end of fusion must lie in the stable zone. While many of these principles are still valid, these have been fine tuned with the advent of modern instrumentation techniques. Particular attention has been paid to reduce the length of fusion at the caudal end in the lumbar spine. Burton *et al*.^22^ defined the caudal foundation vertebra (CFV) on the basis of as the first vertebra at or above the lower end vertebra of the lumbar curve that would become centered over the sacrum after the application of torsional reduction loads.

There has been a tremendous improvement in the instrumentation techniques for correction of scoliosis. The posterior instrumentation technique advanced from two-point fixation with Harrington rods, to segmental fixation with sublaminar wires or hooks, to a combination of pedicle screw-hook fixation, often described as third generation instrumentation. Thoracic pedicle screws are being increasingly used an anchor points, with advantages over hooks. Because of the proximity of the spinal cord, particularly on the concave side of the thoracic curve, it is essential to understand the anatomy of the pedicle, in the deformed vertebrae. The concave side distraction is a more effective mechanism for correction of larger curves, but loses its efficacy as the curve correction approaches a straight line. Rod rotation or lateral translation techniques become more effective for final stages of correction as the curve approaches midline. Burton *et al*. pointed out that in correction of scoliosis, the derotation of the rods should occur in the opposite direction compared to vertebral derotation.^22^ More effective derotation of apical vertebrae can be achieved by using biaxial-pedicle screws. Sublaminar wires, originally developed for neuromuscular scoliosis, have now been established as a cheap but effective alternative for segmental fixation even in idiopathic scoliosis. Basu *et al*.^23^ has presented lateral translation technique, and Bhojraj *et al*.^24^ has presented their experience with sublaminar wiring for AIS in the current focus issues.

Anterior instrumentation may provide better derotation and better correction, but remains limited by the number of segments that can be instrumented through one incision. Whether anterior instrumentation may achieve equivalent correction with smaller segment fusion, and save a motion segment at the caudal end, remains highly debatable. The advantage of anterior surgery for scoliosis is that it may be performed with minimally invasive thoracoscopic approach, with minimal postoperative morbidity. Adult spinal deformity poses a more complex problem. The primary indication is back pain, rather than cosmetic correction. The curves are often large and stiff, and may be associated with sagittal plane deformity or kypohosis. Surgery often requires staged procedures like anterior release, or spinal osteotomy, and a long posterior fusion. Kyphosis associated with global ankylosis requires preoperative planning including size, site, and type of osteotomy, to address restoration of sagittal balance and visual angle correction at the same time.

Spinal deformity correction requires extensive surgical intervention and is prone to develop complications. Thorough pre-operative evaluation of the patient is essential and should include assessment of nutritional status, pulmonary function tests in severe thoracic deformity cases, and presence of other congenital anoimalies in cases of congenital scoliosis. Neurological complication remains the most important risk factor in spinal deformity surgery. Neuromonitoring has made more aggressive correction of the scoliosis safer. SSEP monitoring is easier to perform, and does not interfere with anesthetic technique or use of muscle relaxant, but has occasional false negative events. MEP monitoring is extremely sensitive, but unfortunately has false positive events, which may unnecessarily interrupt surgical procedure. In the current issue Kundnani *et al*.^25^ has discussed sensitivity and specificity with multimodal neuromonitoring on the basis of their experience on 354 consecutive cases. The other important risk in spinal deformity surgery is deep infection, which may occur even after many years.. The congenital scoliosis, post tubercular spinal deformity, adult degenerative scoliosis, pedical morphomatery in scoliosis, advantage of navigation in pedical screw insertion and management of deep infection in scoliosis is presented in forthcoming issue.

Over the past decade, there has been an increasing awareness of evidence-based medicine (EBM). It is being questioned whether surgery for AIS has any significant impact on quality of life (QOL) measures in either the short-term or long term. In the current focus issues there was no review on this aspect, nevertheless this is an important topic, and should be kept in mind before contemplating any surgical intervention.

## WHAT IS THE FUTURE OF SPINAL DEFORMITY SURGERY?

The importance of basic research in understanding of the etiology and pathology of idiopathic scoliosis can not be overemphasized. I hope there will be more reliable methods to predict genetic marker^26^ for early diagnosis and prediction of progression of scoliosis that will be amenable to smaller segment fusion with near complete correction. There may be newer techniques to correct scoliosis using remote-controlled growing rod, gradually over time, so that the deformity may be corrected more completely and safely in patients, while awake. Not much progress has been made in this direction after the initial research published over a decade ago.^27^ Finally, I would like to imagine a day when scoliosis may be corrected, surgically or otherwise, leaving the spine mobile and straight, rather than converting it into a long straight solid fusion mass equivalent to a long bone, with all its long-term consequences.

We thank the authors, reviewers and editorial board of Indian Journal of Orthopaedics to bring symposium on spinal deformity.

## References

[1] Harrington PR (1962). Treatment of scoliosis. Correction and internal fixation by spine instrumentation. J Bone Joint Surg Am.

[2] McMaster MJ, Ohtsuka K (1982). The natural history of congenital scoliosis. A study of two hundred and fifty-one patients. J Bone Joint Surg Am.

[3] Wax JR, Watson WJ, Miller RC, Ingardia CJ, Pinette MG, Cartin A (2008). Prenatal sonographic diagnosis of hemivertebrae: associations and outcomes. J Ultrasound Med.

[4] Lincoln TL (2007). Infantile idiopathic scoliosis. Am J Orthop.

[5] Mehta MH (1972). The rib-vertebra angle in the early diagnosis between resolving and progressive infantile scoliosis. J Bone Joint Surg Br.

[6] Marks DS, Iqbal MJ, Thompson AG, Piggott H (1996). Convex spinal epiphysiodesis in the management of progressive infantile idiopathic scoliosis. Spine (Phila Pa 1976).

[7] Akbarnia BA, Marks DS, Boachie-Adjei O, Thompson AG, Asher MA (2005). Dual growing rod technique for the treatment of progressive early-onset scoliosis: a multicenter study. Spine (Phila Pa 1976).

[8] Pratt RK, Webb JK, Burwell RG, Cummings SL (1999). Luque trolley and convex epiphysiodesis in the management of infantile and juvenile idiopathic scoliosis. Spine (Phila Pa 1976).

[9] Teli M, Lovi A, Brayda-Bruno M (2010). Results of the spine-to-rib-cage distraction in the treatment of early onset scoliosis. Indian J Orthop.

[10] Canavese F, Dimeglio A, D'Amato C, Volpatti D, Granier M, Stebel M (2010). Dorsal arthrodesis in prepubertal New Zealand White rabbits followed to skeletal maturity: Effect on thoracic dimensions, spine growth and neural elements. Indian J Orthop.

[11] Unnikrishnan R, Renjitkumar J, Menon KV (2010). Adolescent idiopathic scoliosis: Retrospective analysis of 235 surgically treated cases. Indian J Orthop.

[12] Wong HK, Tan KJ (2010). The natural history of adolescent idiopathic scoliosis. Indian J Orthop.

[13] Rajasekaran S, Kamath V, Kiran R, Shetty AP (2010). Intraspinal anomalies in scoliosis: An MRI analysis of 177 consecutive scoliosis patients. Indian J Orthop.

[14] Danielsson AJ, Hasserius R, Ohlin A, Nachemson AL (2007). A prospective study of brace treatment versus observation alone in adolescent idiopathic scoliosis: A follow-up mean of 16 years after maturity. Spine (Phila Pa 1976).

[15] Weiss HR (2007). Is there a body of evidence for the treatment of patients with Adolescent Idiopathic Scoliosis (AIS)?. Scoliosis.

[16] Winter RB (2006). Clinical research and outcome analysis for scoliosis surgery: Defining a ‘solid, good result’. J Orthop Surg (Hong Kong).

[17] Wajanavisit W, Woratanarat P, Woratanarat T, Aroonjaruthum K, Kulachote N, Leelapattana W (2010). The evaluation of short fusion in idiopathic scoliosis. Indian J Orthop.

[18] King HA, Moe JH, Bradford DS, Winter RB (1983). The selection of fusion levels in thoracic idiopathic scoliosis. J Bone Joint Surg Am.

[19] Lenke LG, Betz RR, Harms J, Bridwell KH, Clements DH, Lowe TG (2001). Adolescent idiopathic scoliosis: A new classification to determine extent of spinal arthrodesis. J Bone Joint Surg Am.

[20] Goldstein LA (1964). The surgical management of scoliosis. Clin Orthop Relat Res.

[21] Moe JH (1972). Methods of correction and surgical techniques in scoliosis. Orthop Clin North Am.

[22] Burton DC, Asher MA, Lai SM (1999). The selection of fusion levels using torsional correction techniques in the surgical treatment of idiopathic scoliosis. Spine (Phila Pa 1976).

[23] Basu S, Rathinavelu S, Baid P (2010). Posterior scoliosis correction for AIS using side-opening pedicle screw-rod system utilizing the axial translation technique. Indian J Orthop.

[24] Bhojraj SY, Varma RG, Nene AM, Mohite SB (2010). Spinal loop rectangle and sub laminar wiring as a technique for scoliosis correction. Indian J Orthop.

[25] Kundnani V, Zhu L, Tak HH, Wong HK (2010). Multimodal intraoperative neuromonitoring in corrective surgery for AIS: Evaluation of 354 consecutive cases. Indian J Orthop.

[26] Newton PO, Ogilvie JW, Shah SA, Shufflebarger HL (2009). Genetic prognostic testing for adolescent idiopathic scoliosis: Will a genetic test change scoliosis treatment?.

[27] Takaso M, Moriya H, Kitahara H, Minami S, Takahashi K, Isobe K (1998). New remote-controlled growing-rod spinal instrumentation possibly applicable for scoliosis in young children. J Orthop Sci.

